# Plasma soluble lectin-like oxidized low-density lipoprotein receptor-1 acts as a new biomarker for NSTEMI and STEMI patients

**DOI:** 10.4314/ahs.v22i3.37

**Published:** 2022-09

**Authors:** Md Sayed Ali Sheikh

**Affiliations:** Internal Medicine Unit, Cardiology Section, College of Medicine, Jouf University, Sakaka, Saudi Arabia

**Keywords:** Myocardial infarction, sLOX-1, biomarker, H9c2 cells

## Abstract

**Objective:**

The diagnostic significance of plasma soluble lectin-like oxidized low-density lipoprotein receptor-1(sLOX-1) for non-ST segment elevated myocardial infarction (NSTEMI) and ST segment elevated myocardial infarction (STEMI) were explored by this study.

**Methods:**

In this study, 107 acute NSTEMI, 223 acute STEMI and 107 healthy subjects, and hypoxic (1%02) ventricular cardiomyocytes H9c2 were used.

**Results:**

The significantly up-regulated plasma sLOX-1 levels in acute NSTEMI and STEMI patients compared to healthy subjects (p<0.001). Both male and female NSTEMI and STEMI groups had remarkably higher concentrations of plasma sLOX-1 levels than controls (p<0.001). The circulating levels of sLOX-1 expression obviously elevated in elderly aging (60–75 years) than younger aging (30–45 years) both male and female in healthy subjects as well as NSTEMI and STEMI (p<0.001). Altered levels of sLOX-1 in blood plasma revealed a significant discrimination with high sensitivity and specificity between healthy with NSTEMI and STEMI subjects with AUC= 0.916 and AUC= 0.925 respectively. Moreover, LOX-1 levls were highly released from 6hour, 12hour and 18hour hypoxic injured H9c2 cells than normoxic cell (p<0.001), reflected circulating plasma sLOX-1 in AMI patients.

**Conclusion:**

Elevated levels of plasma sLOX-1concentrations might be used as a clinical biomarker for early recognition of NSTEMI and STEMI patients. Multicenter larger scale studies are necessary before use in clinical practice.

## Introduction

Acute myocardial infarction (AMI) is mainly occurred due to critical atherosclerotic occlusion of a coronary artery that leads to significantly decreased circulation to the cardiac tissue subsequently the death of cardiomyocytes followed various fatal arrhythmic events such as ventricular fibrillation or complete heart block and currently is considered as a main issue of premature death across the world with a major socioeconomic burden^1^. Though 20th century tremendous improvement happened in the management of an acute heart attack patient and death rate declined but still AMI incidence rate is increasing due to lifestyle changes, workload stress, overconsumption of cholesterol containing foods, lack of physical exercise and sleeping disturbances as well as increasing aging people with more uncontrolled diabetes and hypertension^2^. Early diagnosis and rapid primary percutaneous coronary intervention remarkably decreased AMI induced life threatening complications and outstandingly increased survival rate. Currently, up-regulated high sensitive cardiac troponins (cTnT, cTnI) assays are the only recommended as a biochemical marker for initial evaluation of suspected acute myocardial infarction patients in clinical practice all over the world. However, plasma cardiac troponins are also significantly up-regulated in severe renal and heart failure patients. Besides, in NSTEMI patients' cardiac troponins are usually not increased in earlier stage. Therefore, to discover a new more appropriate clinical biomarker for early detection of AMI patients are demanded by the clinical cardiologist^3^,^4^.

Lectin-like oxidized low-density lipoprotein receptor-1 (LOX-1) an OLR1 gene product was initially recognized as an essential receptor for atherogenic oxidized low-density lipoprotein (ox-LDL) in endothelial cells. Several studies reported, circulating ox-LDL expressions were highly up-regulated in various atherosclerotic cardiovascular disease conditions^5^,^6^. Intimal smooth muscle cells and lipid-laden macrophages are the major sources of LOX-1 expression. Furthermore, LOX-1 is also released by cardiomyocytes, fibroblasts, adipocytes, platelets, neurons, lungs and the placenta^7^,^8^.

Soluble LOX-1 has usually been detected in serum and plasma, and sLOX-1concentrations have been directly illustrated of the LOX-1 expression^9^. Coronary atherosclerosis is a chronic inflammatory disorder and the most susceptible at the curved or branching area of the coronary arteries^10^. It has been well established that LOX-1 plays a fundamental task in Ox-LDL induced vascular inflammation, endothelial dysfunction, apoptosis of vascular smooth muscle as well as increased production of various matrix metalloproteinases resulting formation and rupture of an atherosclerotic plaque within a coronary artery. Moreover, LOX-1 is also critically involved in thrombus formation after plaque rupture that occluded coronary arteries and developed acute heart attack by association with activated platelets^7^,^11^. A very recent study has been reported that LOX-1 expression was significantly elevated in intramyocardial and epicardial coronary arteries patients undergoing coronary bypass surgery^12^. The LOX-1 induced cardiac myocytes apoptosis and myocardial cellular injuries have been involved through p38 mitogen-activated protein kinase pathway^13^,^14^.

Balın M et al. demonstrated that circulating soluble LOX-1 levels were significantly associated with proximal/mid segment of the left anterior descending artery (LAD) lesions. They suggested elevated circulating LOX-1 concentrations might be used as a helpful biomarker for higher risk of stable coronary artery plaque rupture patients^15^. In our previous study, we found the serum levels of LOX-1 were significantly increased in stable coronary artery disease patients and strong association with metabolic syndrome^8^. Circulating sLOX-1 was markedly elevated in acute coronary syndrome (ACS) patients as compared with non- ACS patient, considered as a reliable specific marker for the recognition of ACS patients^11^, ^16^. However, the expressions of plasma of sLOX-1 levels in acute NSTEMI and STEMI patients have not been fully elucidated. Therefore, current study evaluated the diagnostic role of circulating sLOX-1 for early detection of acute NSTEMI and STEMI patients and their expression pattern among different ages and gender. Besides, expressions of LOX-1 from healthy and hypoxic injured cardiomyocytes were also investigated.

## Materials and methods

### Study subjects

In total, 437 subjects were participated in this current clinical study, acute STEMI patients were 223 among them 127 were males and 96 were females with average age (62.16±7.95) years, 107 acute NSTEMI patients with males 66 and females 41 with mean age were (62.18±7.80) years, 107 age and gender well matched healthy volunteers amongst 54 were males and 53 were females and their average age (62.18±7.80) years. All the study subjects were recruited in between January 2016 to August 2018 from the first affiliated Xiangya hospital cardiovascular medicine unit and health center of Central south university. Acute STEMI and NSTEMI were categorized by following the American College of Cardiology, the American Heart Association, the World Heart Federation and the European Society of Cardiology clinical practice guidelines.^2^ Healthy volunteers were free from cardiovascular or cerebrovascular and any form of acute or chronic infections or inflammatory diseases. This study was conducted by the following revised 2013 Declaration of Helsinki (World Medical Association) human experimental principles and had been approved by the Ethics Committee of Xiangya Hospital, Central South University. Before the experiments from all the study participants written informed consent were taken. Five ml peripheral blood samples were collected from NSTEMI and STEMI patients (ischemic chest pain less than12hours) and also from healthy participations from an antecubital vein in EDTA tubes. Immediately after samples collection blood were centrifuged at 15,000 rpm for 10 minutes at 4°C, subsequently supernatants were collected into new tubes and re-centrifuged for another 5 minutes at 10,000 rpm to collect debris and cell free pure plasma, and kept at -80°C for further analysis. The enzyme-linked immunosorbent assay (ELISA) method used for the measurement of plasma sLOX-1 levels (BioSource Inc, CA, USA). Moreover, the concentrations of Triglycerides (TG), Low-density lipoprotein (LDL), Total cholesterol (TC), High-density lipoprotein (HDL) and high sensitive C-reactive protein (hs-CRP) were measured with Roche (USA) automated chemistry analyzer kits.

### Hypoxic model of H9c2 cells

Ventricular heart (rat) originated cardiomycytes H9c2 harvested in 12 cell culture well plates by adding ten percent fetal bovine serum in Dulbecco's modified Eagle's medium (DMEM, Gibco, Waltham, USA) in a cell model incubator with constant maintained of 95% air, 5% CO2 and 37°C temperature. H9c2 cells hypoxia models were generatedby using a hypoxic model incubator (Model 3131; Forma Scientific, OH, USA) with serum and glucose free DMEM for 6, 12 and 18 hours at 37°C in an atmosphere containing 95% Nitrogen, 5% CO2 and 1% Oxygen.

### Isolation of RNA and Real time PCR

Total RNA was extracted from normal and Hypoxic H9c2 cells by using TRIzol cell breaking down chemical reagent (Invitrogen, CA, USA) following the company's guidelines. Pure isolated RNA was transferred into EP tubes and stored at-80°C for future analysis. Quality of RNA was determined through NanoDrop ND-1000 spectrophotometer. Subsequently RNA samples were converted into cDNA. Finally, quantification of LOX-1 mRNA was measured with SYBR Green Master Mix PCR reagents (Takara, Dalian, China) through Real-Time PCR System and SDS 2.4 software (Applied Biosystems, USA). All the samples were examined three times and GAPDH used as standard inner control.

### Analysis of statistical data

SPSS version 19 was applied for the measurement of all statistical data analysis. Independent samples t test, one-way and two-way ANOVA, LSD and bonferroni posthoc test, weight case, descriptive study, Chi-square tests were carried out for comparisons among different groups with variables as per required. All figures were constructed with Prism windows 6 version. The clinical diagnostic importance of sLOX-1 was evaluated by using areas under the curve (AUC) the receiver operator characteristic (ROC) curve. Sample sizes were determined by using GPower method. Less than 0.05 accepted as a significant P value.

## Results

### Baseline characteristics of the participants

Among 107 patients with NSTEMI, 66 were males and 41 females, age ranged from 30–75 years (62.18±7.80), 223 STEMI patients among them 127 were males and 96 females (30–75 years) with mean age (62.16±7.95) years, Age and sex matched 107 were healthy controls, 54 were males and 53 females and their age between 30–75 years (62.18±7.80). Baseline characteristics for all the participants (n=437) were shown in table-1. SBP, DBP, TC, HDL, TG, LDL and hs-CRP levels were significantly different between healthy subjects with Non-STEMI and STEMI (P < 0.001) but with Non-STEMI and STEMI were not statistically significant (Table 1).

**Table 1:** Baseline characteristics of the enrolled subjects

Baseline data	Healthy participants (n=107)	Non- STEMI (n=107)	STEMI (n=223)	P_1_	P_2_	P_3_
Male/Female	54/53	66/41	127/96	0.130	0.289	0.474
Age (mean ± st.d) years	62.18±7.80	63.76±7.16	62.16±7.95	0.295	1.000	0.183
Body mass index, (kg/m2)	21.87±2.46	22.38±3.25	22.81±3.26	0.440	0.21	0.403
Current smoker	60.74%	63.22%	65.02%	0.778	0.465	0.807
Type-2DM	0	26.16%	28.25%	0	0	0.793
Hypertension	0	65.42%	66.81%	0	0	0.805
Lipid disorders	0	73.83%	71.30%	0	0	0.695
Systolic BP(mmHg)	125.70±7.8	131.74±17.1	133.40±17.8	0.011	≤0.001	0.629
Diastolic BP(mmHg)	74.47±6.1	79.01±8.87	81.12±9.78	≤0.001	≤0.001	0.106
Serum TG (mmol/L)	1.311±0.24	1.414±0.72	1.414±0.72	0.481	0.092	0.731
Serum TC(mmol/L)	4.36±0.24	6.30±3.06	6.43±2.60	≤0.001	≤0.001	0.879
Serum HDL(mmol/L)	1.14±0.41	0.82±0.39	0.80±0.38	0.000	≤0.001	0.790
Serum LDL(mmol/L)	2.18±0.54	3.43±1.87	3.67±1.94	≤0.001	≤0.001	0.211
hs-C-reactive protein (mg/L)	2.69±1.53	16.74±7.46	18.01±9.14	≤0.001	≤0.001	0.323
Left ventricular EF%	63.86±8.15	58.57±5.61	57.52±6.87	≤0.001	≤0.001	0.392

DM; Diabetes mellitus, BP; blood pressure, HDL; High-density lipoprotein, TG; Triglycerides, LDL; Low-density lipoprotein, TC; Total cholesterol, EF; Ejection fraction. P1 (Controls and NSTEMI patients), P2 (Controls and STEMI patients), and P3 (NSTEMI and STEMI).

### Circulating sLOX-1 expressions pattern in acute myocardial infarction patients

Up-regulated expressions of circulating plasma sLOX-1 were observed in patients with NSTEMI and STEMI than controls (p<0.001) but the differences were not significant. In both NSTEMI and STEMI groups' plasma sLOX-1 levels were up-regulated by 5.56 and 5.71 folds respectively compared to healthy controls (Figure-1A). Moreover, circulating sLOX-1 concentrations were remarkably increased in both male and female NSTEMI and STEMI patients (p<0.001) than healthy male and female but among NSTEMI and STEMI male and female groups were not significant (Figure- 1B).

**Figure:**
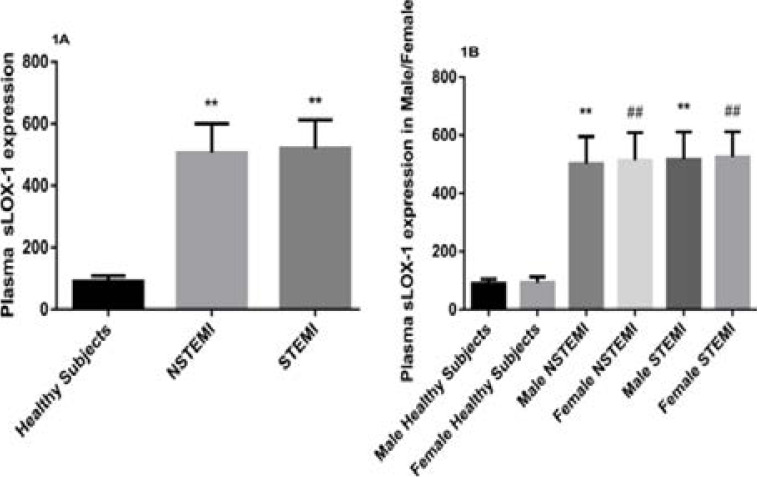
**Figure 1A:** Expression of plasma sLOX-1 levels in healthy subjects versus NSTEMI and STEMI (p<0.001). **Figure 1B:** Circulating sLOX-1 expression levels in plasma of Male and Female Gender among healthy, NSTEMI and STEMI subjects (p<0.001).

### Plasma sLOX-1 expressions in Male and Female among different age groups of Healthy and AMI subjects

Circulating sLOX-1 levels were gradually increased with aging in both male and female of healthy and AMI subjects. Plasma sLOX-1 concentrations were significantly up-regulated in (60–75years) healthy male and female groups than (30–45years) male and female healthy subjects (p<0.001) respectively (Figure-2A). In NSTEMI, expressions of circulating sLOX-1 were prominently increased in both male and female in (60–75years) age groups compared with male and female (30–45years) age groups respectively(p<0.001) (Figure-2B). Moreover, plasma levels of sLOX-1 in (60–75years) groups male and female STEMI patients were remarkably higher than male and female of (30–45years) age groups (p<0.001) respectively, (Figure-2C).

**Figure:**
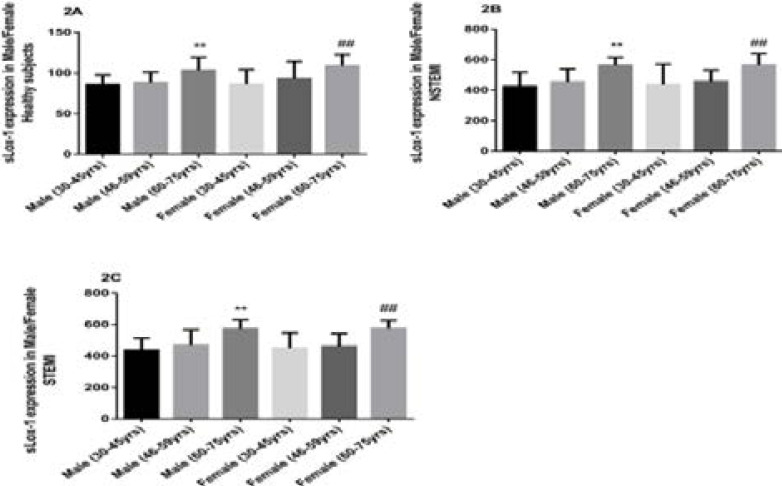
**Figure 2A:** Plasma sLOX-1 expression in different age groups of both male and female in healthy subjects, male and female (30–45years) vs male and female (60–75years) (p<0.001). **Figure 2A:** Expression of plasma sLOX-1 levels in NSTEMI patients within different ages and genders, male and female (30–45years) groups versus male and female (60–75years) (p<0.001). **Figure 3A:** Circulating sLOX-1 concentrations in STEMI patients, (30–45years) male and female genders versus (60–75years) male and female genders (p<0.001).

### Diagnostic significance of Plasma sLOX-1 levels in AMI patients

**Figure 3:**
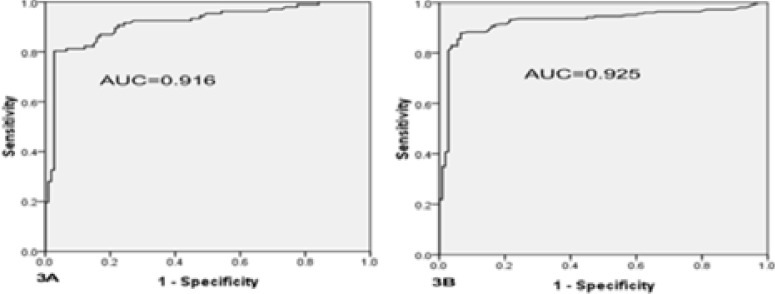
Diagnostic importance of plasma sLOX-1 for acute NSTEMI and STEMI patients were evaluated with Receiver operating characteristic (ROC) curves analyses: Figure: 3A, Healthy controls versus NSTEMI (AUC 0.916) (p<0.001). **Figure: 3B**, Healthy subjects and acute STEMI (AUC 0.925) (p<0.001).

Diagnostic role of circulating sLOX-1for NSTEMI and STEMI patients were explored by ROC curve analysis. Plasma sLOX-1 levels showed a strong discrimination among healthy and NSTMI patients (AUC 0.916) (Figure-3A) and also between healthy subjects with STEMI patients (AUC 0.925) (Figure-3B) and they were statistically highly significant (p<0.001). However, ROC curve analyses were unable to differentiate within the patients of STEMI and NSTEMI, and statistically also non-significant. These results suggested increased levels of blood plasma sLOX-1 may be used as a new sensitivity biomarker for early evaluation of AMI patients.

### The association of circulatory sLOX-1 concentrations with other clinical parameters

**Figure 4:**
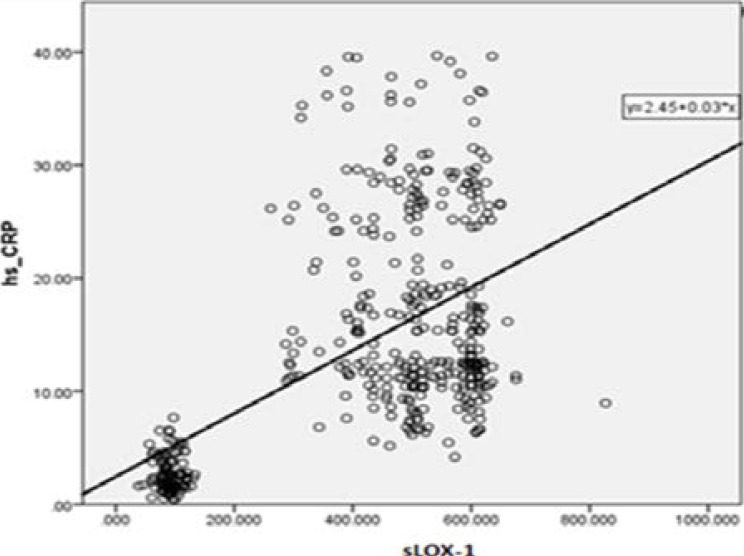
Correlation among sLOX-1 and hs-CRP in AMI patients.

The relationship between plasma sLOX-1 and other clinical characteristics was evaluated and noticed that concentrations of sLOX-1 were moderately associated with hs-CRP (Figure-4, table- 2).

**Table 2:** Clinical markers and sLOX-1

Clinical parameters	r-vale	P value
hs-CRP	r= 0.56	< 0.001
LVEF	r = -0.31	< 0.001
SBP	r = 0.19	< 0.001
DBP	r = 0.26	< 0.001
TC	r = 0.33	<0.001
HDL	r = -0.31	< 0.001
LDL	r = 0.34	< 0.001

### LOX-1 expression in Healthy and Hypoxic H9c2 cells

**Figure 5:**
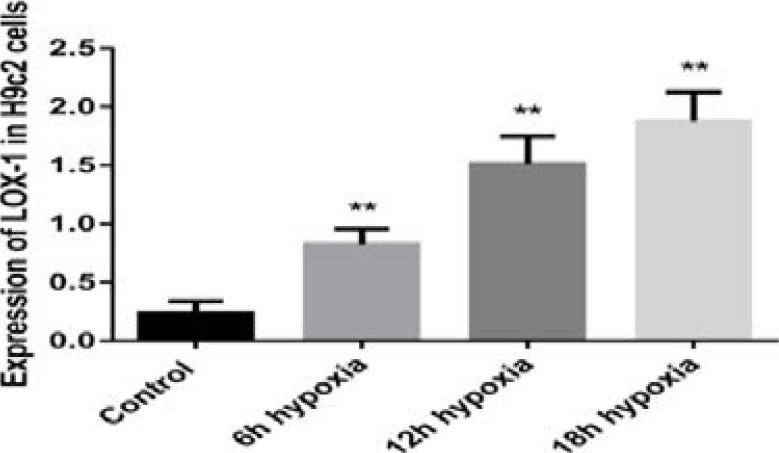
LOX-1 expression patterns in Healthy and Hypoxic H9c2 cells. Control with 6hour, 12hour and 18hour hypoxic challenge H9c2 (p<0.001). Within 6hour, 12hour and 18hour hypoxic H9c2 cells (p<0.001).

Expressions of LOX-1 mRNA levels were remarkably increased in 6hour (3.41 fold), 12hour (6.28 fold), and 18hour (7.78 fold) in hypoxic exposed H9c2 cells than normal cells (p<0.001), respectively. The LOX-1concentrations were gradually increased in proportion with increasing hypoxic time (Figure 5). Moreover, expression of LOX-1 among 6hour,12hour and 18hour in hypoxic insults H9c2 cells were obviously significant (p<0.001). These results strongly indicated that LOX-1 was released from cardiomyocytes injury and significantly linked with myocardial infarction.

## Discussion

Coronary atherosclerotic plaque rupture followed by thrombus formation associated with coronary artery spasm is the critical step for the development of myocardial infarction. LOX-1 has taken a major role in the pathogenesis of atherosclerosis and it is also significantly expressed from erosion of atherosclerotic plaques as well as surrounding tissues^17^. LOX-1 up-regulated various inflammatory pathways including NF-KB, mitogen activated protein kinase and released cytokines such as TNF-α, IL-1, IL-8, endothelin, monocyte chemoattractant protein, and platelet derived growth factor. Furthermore, LOX-1 through binding with OX-LDL increased ROS production by enhancing NADPH oxidase, oxidative induced markers (3-NT4-HNE) and decreasing eNOS activity, elevated cellular apoptosis and cell death through over expression of caspase-3, caspase-9 and inhibition of anti-apoptic protein BCL2 via PI3K/Akt/eNOS signaling^18–20^.

In this clinical study, plasma sLOX-1 expressions were determined in acute NSTEMI and STEMI patients. The present study has shown circulating sLOX-1 levels significantly up-regulated in both NSTEMI and STEMI patients than the healthy controls. Female NSTEMI and STEMI patients had higher levels of plasma sLOX-1 expression as compared with male NSTEMI and STEMI patients, but were not statistically significant.

Noriaki et al. noticed serum soluble LOX-1 concentrations remarkably up-regulated in patients with acute coronary syndrome (ACS) than non- ACS subjects^11^. A very recent study reported plasma sLOX-1 expressions were significantly higher in STEMI patients and positively linked with L5, sLOX-1 may be a valuable diagnostic marker for AMI patients^21^. A multicenter cohort study confirmed the expressions of soluble serum LOX-1 prominently elevated in CAD patients treated with primary percutaneous coronary intervention (PCI), they also noticed higher serum sLOX-1concentrations were directly associated with negative impact on cardiovascular and cerebrovascular events (MACCEs), perhaps sLOX-1 considered as a diagnostic and prognostic biomarker^22^. Circulating soluble LOX-1 expressions noticeably increased in patients with angina pectoris and closely linked with proximal and middle segments of the left anterior descending coronary artery lesions (LAD), moreover, in our previous research, we also demonstrated serum LOX-1 levels had significantly higher in stable coronary artery disease and metabolic syndrome patients than healthy subjects^10^,^8^. It has been recognized that circulating LOX-1 concentrations were obviously up-regulated in dyslipidemic, hypertensive, diabetes and metabolic syndrome patients^17^. To the best of knowledge, current study first time explored the plasma sLOX-1 concentrations were slowly up-regulated along with increasing the age of both healthy and AMI subjects. Besides, expressions of circulating sLOX-1 were considerably higher (p<0.001) in male and female healthy and NSTEMI and STEMI elderly (60–75years) groups than younger age (30–45years) groups.

Plasma sLOX-1 diagnostic importance for NSTEMI and STEMI patients were evaluated through ROC curve analysis, and found that expressions of sLOX-1 were able to clearly differentiate between acute NSTEMI and STEMI patients and healthy volunteers with higher sensitivity and specificity and significant AUC of 0.916 and AUC of 0.925 respectively, however, AUC values were not significant among acute NSTEMI and STEMI groups. These results strongly indicated that plasma sLOX-1 can be considered a potential sensitive novel useful investigative marker for early detection of all categories non-STEMI and ST-elevated MI patients. This study also prominently supported by other studies^11^, ^15^^,22^.

The very important finding of the current research in the cellular experiment was that LOX-1 expressions were prominently higher in 6hour, 12hour and 18hour hypoxic induced H9c2 cells than control cells. These findings suggested that LOX-1 significantly expressed from cardiac tissues during hypoxic injury and it also partially supported by other studies^13^^, 23–24^. A research study reported that LOX-1 prominently expressed from hypoxia induced rat ventricular derived H9c2 cells through up-regulation of NOX-2 and NOX-4 proteins, but oxidative stress remarkably reduced by the lockdown of the LOX-1 gene^25^. Several research studies were reported that TG, LDL, HDL, TC and hs-CRP had positively correlated with atherosclerosis and various cardiovascular events including cerebral infarction and acute coronary syndrome^11^, ^26–30^. In this study also found TC, HDL, LDL and hs-CRP levels were significantly changed between healthy controls with NSTEMI and STEMI patients but within NSTEMI and STEMI subjects were not significant. An inflammatory marker of high sensitive C-reactive protein and its association with sLOX-1 levels will be needed more future study to explore their underline molecular pathways. Moreover, to reduce the possible bias of the results in this study properly matched age and sex healthy and diseased subjects were chosen. Within 12hours of chest pain from disease groups and overnight fasting for control groups blood samples were collected and immediately processed to get fresh plasma. Every sample was examined by three times during ELISA protocols and at least more than 3-fold changes compared with controls that were used during data analysis.

## Conclusion

Elevated plasma soluble LOX-1 level is the major modifiable risk factor for ischemic heart disease and could be considered as a suitable diagnostic biomarker for acute myocardial infarction patients but further larger clinical studies are required.
